# Method to improve the survival of night-swarming mayflies near bridges in areas of distracting light pollution

**DOI:** 10.1098/rsos.171166

**Published:** 2017-11-15

**Authors:** Ádám Egri, Dénes Száz, Alexandra Farkas, Ádám Pereszlényi, Gábor Horváth, György Kriska

**Affiliations:** 1 MTA Centre for Ecological Research, Danube Research Institute, 1113 Budapest, Karolina út 29-31, Hungary; 2 Environmental Optics Laboratory, Department of Biological Physics, Physical Institute, ELTE Eötvös Loránd University, 1117 Budapest, Pázmány sétány 1, Hungary; 3 Group for Methodology in Biology Teaching, Biological Institute, ELTE Eötvös Loránd University, 1117 Budapest, Pázmány sétány 1, Hungary; 4 Department of Zoology, Hungarian Natural History Museum, Bird Collection, 1083 Budapest, Ludovika tér 2-6, Hungary

**Keywords:** *Ephoron virgo*, ecological light traps, phototaxis, nature conservation, light pollution, visual ecology

## Abstract

Numerous negative ecological effects of urban lighting have been identified during the last decades. In spite of the development of lighting technologies, the detrimental effect of this form of light pollution has not declined. Several insect species are affected including the night-swarming mayfly *Ephoron virgo*: when encountering bridges during their mass swarming, these mayflies often fall victim to artificial lighting. We show a simple method for the conservation of these mayflies exploiting their positive phototaxis. With downstream-facing light-emitting diode beacon lights above two tributaries of the river Danube, we managed to guide egg-laying females to the water and prevent them from perishing outside the river near urban lights. By means of measuring the mayfly outflow from the river as a function of time and the on/off state of the beacons, we showed that the number of mayflies exiting the river's area was practically zero when our beacons were operating. Tributaries could be the sources of mayfly recolonization in case of water quality degradation of large rivers. The protection of mayfly populations in small rivers and safeguarding their aggregation and oviposition sites is therefore important.

## 1. Introduction

Artificial night-time lighting technologies are expanding worldwide [1] and spectral characteristics of the new light sources are becoming increasingly apparent [2]. The severe negative ecological impacts of urban light pollution on animal behaviour [3–6] and wild plants [7] are becoming more widely investigated in terrestrial ecosystems, marine habitats and coastlines [8–10]. Owing to their improved energy efficiency, light-emitting diodes (LEDs) are spreading rapidly [11], but their negative impacts on the environment [12,13] and public health [14,15] came to light one after the other. Artificial light sources are one of the main causes of ecological traps [16–18], and polarized light pollution caused by strongly and horizontally polarized light reflected from artificial shiny dark surfaces (e.g. asphalt roads, solar panels, glass buildings, black plastic sheetings or gravestones) also have disruptive effects, particularly on polarotactic aquatic insects [19–21].

**Figure 1.**
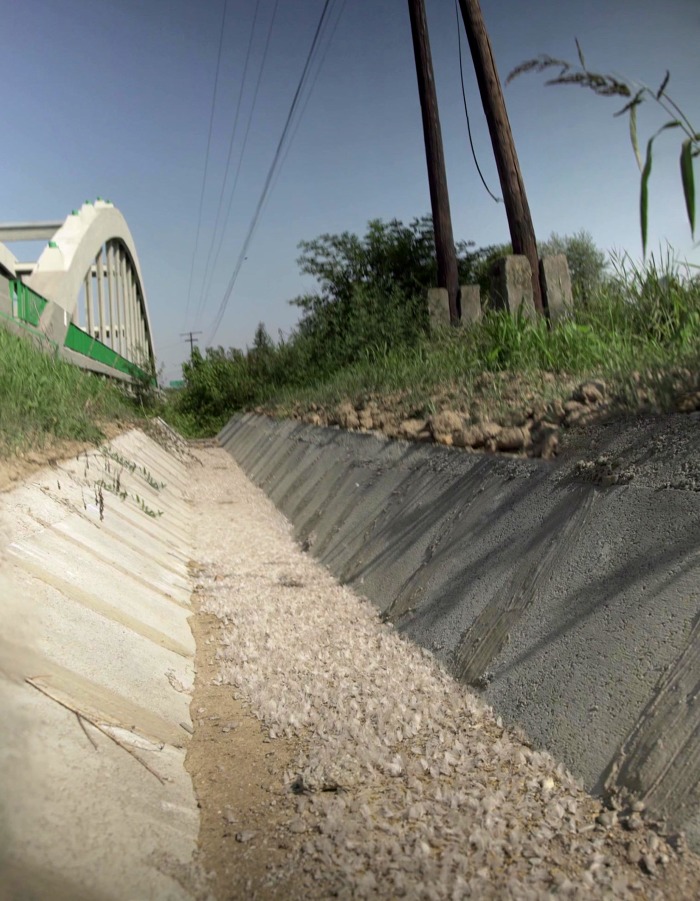
Thousands of female *Ephoron virgo* carcasses covering the bottom of the ditch under street lamp R1 (figure 2*d*) at Rábahídvég in the morning of 6 August 2015. As confirmed recently, the compensatory swarm of *E. virgo* consists of only adult females [22].

Several anecdotes, press reports and case studies have showed that illuminated boats and lamplit bridges overarching a river are major threats to nocturnal mayfly populations, like *Ephoron virgo* (Olivier, 1791) [22]. Huge mayfly swarms around illuminating street lamps were observed on the river Danube [22–25], Mississippi [26], Rhine [27], Main [28], at the Ardahan bridge on Kura river [29], bridge of Tudela on Ebro river [30] and bridge of Route 462 over the Susquehanna river, Pennsylvania [31]. According to Száz *et al*. [22], the complex ecological trap of lamplit bridges can be explained by the interaction of polarized and the regular unpolarized ecological light pollution: First, the bridge forms an optical barrier which interrupts the upstream-directed compensatory flight of night-swarming mayfly females (e.g. *E. virgo*) by disrupting the strongly and horizontally polarized signature of the river surface. Then the disrupted mayflies get attracted towards the unpolarized bridge lights and form huge and vertiginous swarms around them, due to their positive phototaxis. Finally, instead of laying their eggs into the river, the females oviposit on the horizontally polarizing asphalt road of the bridge, due to their positive polarotaxis [19,21]. As a result of the whole process, huge piles of female carcasses and eggs cover the bridge at the end of the swarming (see figure 1 of this paper and fig. 2 of Száz *et al*. [22]). Eyewitnesses also reported that the visibility can be greatly reduced by the dense swarms of flying *Hexagenia bilineata* (Say 1824) mayflies and their corpses can make the roads and decks slippery and dangerous [26].

A number of strategies have been proposed to minimize the ecological light pollution, but changes in the spectral composition and spatio-temporal pattern of night-time lighting are also recognized as having ecological effects [32–34]. Maintaining and increasing the proportion of naturally illuminated areas are likely to be the most effective way for reducing ecological light pollution, but this can lead to conflicts due to social and economic objectives, demands and regulations [32]. For eliminating the mentioned ecological trap of night-swarming mayflies a complete removal or provisional deactivation of bridge-lamps would be the simplest solution [22], or the concerned road-sections and bridges could be blocked temporarily. As a notable positive example, the art deco lights on the Route 462 in Pennsylvania were hurriedly turned off when huge masses of nocturnal mayflies emerged from the Susquehanna river and left these lamps dark for their whole swarming activity [35]. But in most cases, the bridge-lighting is necessary for traffic safety [22].

Száz *et al*. [22] studied the polarotaxis and the attraction of *E. virgo* mayflies to 100% horizontally polarized and unpolarized light sources of equal intensity and found that horizontally polarized light attracted 5–10 times more mayflies than the unpolarized one. This fact provided inspiration for applying protection lamps emitting horizontally polarized light, which can attract and keep the night-swarming mayflies close to the river surface, so that they can lay their eggs into the water. However, Farkas *et al*. [25] showed that horizontally polarized light was only 1.2 times more attractive for *E. virgo* than the unpolarized one, when the polarized light source was 0.43 times as dim as than the unpolarized one. Consequently, it was not worth applying horizontally polarized light for attracting mayflies because a polarizing filter at least halves the intensity of a light source and the expected mayfly attraction is then not significantly greater compared to the attraction to the original, non-filtered unpolarized light.

In this study, we show a tested simple method aimed to increase the survival of ovipositing mayflies near illuminated bridges. This conservation remedy uses unpolarized beacon lights above the river surface to attract egg-laying female mayflies by guiding them to the water and preventing them from perishing outside the river near urban light sources.

## 2. Material and methods

**Figure 2.**
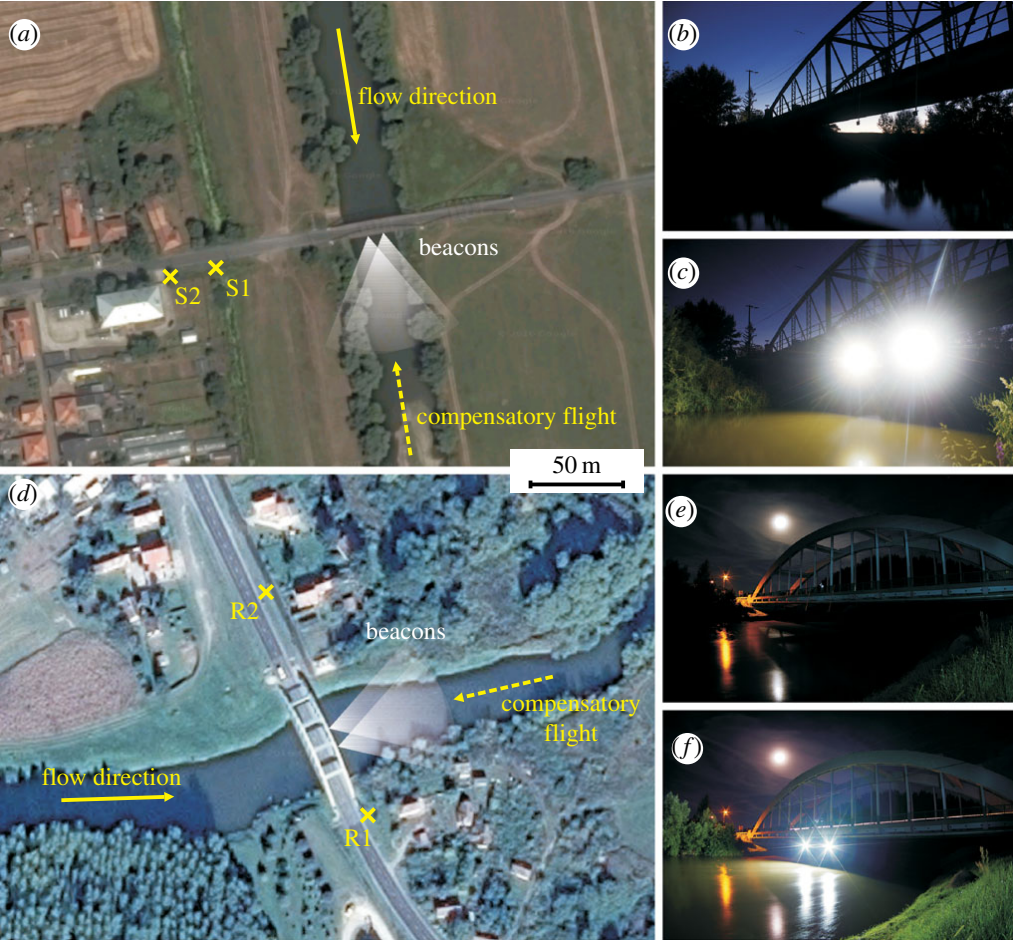
Sites of our field experiments. (*a*) Satellite image of the experimental location at Salka (Slovakia). S1 and S2 are the positions of the street lamps standing nearest to the river Ipoly. (*b*,*c*) The bridge over the river Ipoly at Salka with the beacons switched off (*b*) and on (*c*). (*d*) Satellite image of the experimental location at Rábahídvég (Hungary). R1 and R2 are the positions of the street lamps standing nearest to the river Rába. (*e*, *f*) The bridge of Rábahídvég with the beacons switched off (*e*) and on (*f*). The satellite images originate from *maps.google.com*.

Based on a preliminary test (6 August 2015), we performed two identical experiments in 2016 on two tributaries of the river Danube. The swarming period of *E. virgo* lasts approximately one week at a given site, but the intensity of the swarming is rather stochastic which means that the number of swarming mayflies cannot be easily predicted from swarming events of the previous evenings. Thus, the experimenters had to stay in the field 2–3 days long for one successful measurement. Our first experiment was done on 9 August near Salka (Slovakia, 47.8862° N, 18.7629° E) on the river Ipoly forming the border between Slovakia and Hungary (figure 2*a–c*). Five days later, in the evening of 14 August, about 200 km away, we performed the second experiment at the bridge of the village Rábahídvég (Hungary, 47.0635° N, 16.7458° E) overarching the river Rába (figure 2*d–f*), where the preliminary test had also been carried out the year before. Every summer the inhabitants of these localities observed that huge masses of *Ephoron virgo* were attracted by urban lights near the river, close to the bridge of the village, and the lured mayflies got exhausted and devastated under the street lamps (figure 1), as it has previously been observed by Száz *et al*. [22] on the river Danube in Hungary, and by Kazanci and Türkmen [29] on the Kura river in Turkey.

**Figure 3.**
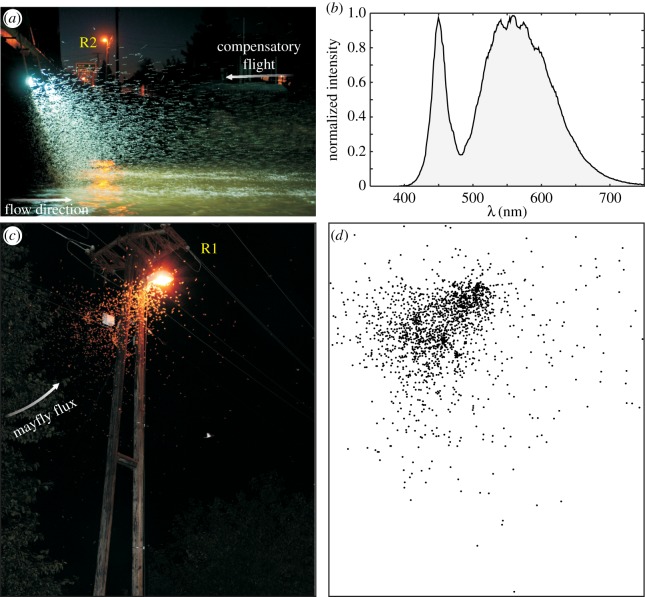
Mayfly swarms around the light sources in the experiments. (*a*) Photograph of the mayfly cloud formed around the beacons attached to the middle of the bridge on river Rába at 21.13 (UTC + 2 h). R2 is a street lamp marked in figure 2*d*. (*b*) Emission spectrum of the beacons. (*c*) Street lamp R1 with swarming *E. virgo* mayflies appeared after switching off the beacons on the bridge at Rábahídvég for the first time (21.16, UTC + 2 h). (*d*) Positions of the 1620 recognized *E. virgo* specimens on image (*c*).

At each experimental site before sunset, two battery-powered, 50 W, 4000 Lumen, cool white (6000 Kelvin) LED flood lights (hereinafter: beacons) were hung down from the bridge rail 3 m beneath the asphalt level, facing downstream (figure 2). According to figure 3*b*, the emission spectrum of our portable LED light sources measured with an Ocean Optics STS-VIS spectrometer was bimodal with peaks near 450 and 550 nm. The beacons were 8 m apart above the river's midline. At the same time, two tripod-equipped DSLR cameras were aimed at the two street lamps being closest to the bridge (figure 2*a*,*d*). As the dusk went by, we monitored and checked the river with flashlights and turned on the beacons when the first mayfly imagoes appeared. Later, when the mayfly swarm began to grow significantly around the beacons, we started to take photographs simultaneously with the two cameras, at a rate of approximately three images per minute per camera. We took 276 and 352 photographs at the first (Ipoly) and second (Rába) experimental site, respectively.

The swarming lasted about 1 h each day. During that time, we switched off the beacons three times for 3–6 min in order to demonstrate that the mayflies leave the river and get trapped at the street lamps in huge masses only when the beacons were switched off. Later, the photographs of the street lamps were evaluated by counting the attracted mayflies on the images manually, thus we used the numbers of mayflies attracted to the street lamps on the shore as a measure of mayfly destruction. This choice was reasonable, since the mayflies that were once attracted to the street lamps, inevitably perished on the asphalt road or in the ditch beneath. Figure 3*c* shows an example photograph with 1620 counted *E. virgo* specimens (figure 3*d*). However, we used flash and the street lamp illuminated the surrounding mayflies as well, thus we could only locate a subgroup of them and unavoidably overlooked the darkened individuals. On the other hand, the sometimes crowded mayflies overlapped each other. Consequently, we underestimated the exact numbers of the attracted mayflies, but it is not a problem since we were interested in the dynamics of the mayfly outflow from the river, and rather than the exact numbers of specimens.

## 3. Results

**Figure 4.**
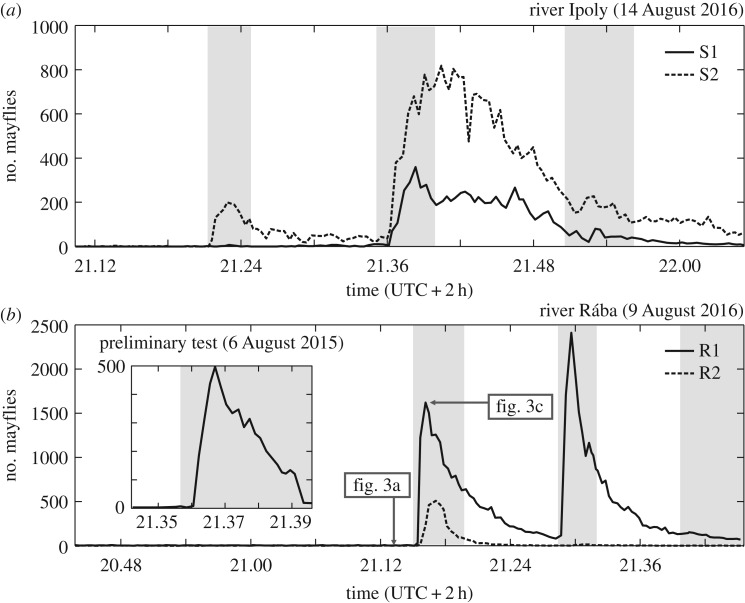
Numbers of detected mayflies around the studied street lamps as a function of time. Solid and dashed lines represent the curves for the closest (S1, R1) and the second closest (S2, R2) functioning street lamps from the midline of (*a*) river Ipoly and (*b*) river Rába. The inset in (*b*) shows the numbers of mayflies at street lamp R1 as a function of time during the preliminary test.

We collected data from two different sites and obtained practically the same results in both cases. During the evaluation process, we manually identified 32 498 and 47 072 *E. virgo* specimens on the photographs taken at the rivers Ipoly and Rába, respectively. Figure 4 shows the number of mayflies around the two nearest street lamps (figure 2*a*,*d*) as a function of time in the field experiments. Grey areas represent the time intervals when the beacons were switched off. The most glaring characteristic of the curves is that the mayfly numbers increase only right after switching off the beacons. This means that the arriving mayflies did not leave the river until the beacons were switched on, although huge masses of females were present in front of the lights under the bridge (figure 3*a*). When the beacons were switched off, the mayfly swarm left the river and approached the street lamps standing next to the road (figure 3*c*,*d*; electronic supplementary material, video M1). Once the mayflies overran the street lamps their number decreased with time, as seen in the curves of figure 4. The reason for this was twofold: (i) As time passed the mayflies got exhausted and dropped into the ditch under the street lamps (figure 1). (ii) A minor portion of the mayflies became aware of the nearest street lamps nearby and their lamp-to-lamp approaches led to a decrease in their numbers at the initial lamp.

As figure 4 shows, the third switch-off did not elicit a sudden increase in the number of females around the closest street lamps at both experimental locations. The reason for the lack of the peak in the curves is related to the end of the swarming. After 21.40 and 21.30 at Ipoly and Rába, respectively, only very few *E. virgo* specimens accumulated at the operating beacons and the third switch-off did not result in high mayfly flux towards the street lamps. Differences between the mayfly numbers near the two closest street lamps are also noticeable at both experimental locations. At river Ipoly, the light intensity of the closest lamp S1 was significantly lower than that of S2, thus the former attracted much fewer mayflies. In the case of the river Rába, the intensities of the two closest street lamps R1 and R2 were equal. Here, R1 attracted significantly more specimens than R2, because R1 was closer to the river (figure 2*d*).

The inset in figure 4*b* represents a 5-min-long measurement we performed 1 year before, in 2015, as a preliminary test mentioned at the beginning of the Material and methods. As this inset shows, we experienced the same phenomenon: Female mayflies arriving in their compensatory flight left the river and got attracted to street lamp R1 (figure 2*d*) only when we switched off the beacons.

Although the curves in figure 4 are informative, we also created videos for the visual demonstration of our mayfly-conserving method (electronic supplementary material, videos M1–M3). Electronic supplementary material, video M1 shows the most significant parts of our experiments. The scenes of M1 were recorded during the preliminary test in 2015 and the type of the power LEDs composing the lights were different from those of the real experiments (electronic supplementary material, videos M2 and M3). Electronic supplementary material, videos M2 and M3 show scenes from the experiments conducted on the river Ipoly and Rába, respectively.

All three videos contain an additional phenomenon, the knowledge of which cannot be obtained merely from the curves of figure 4: If the size of the mayfly cloud in front of the beacons exceeds a critical value, the mayflies at the bottom of the cloud become stuck on the water surface. Thus, the beacons guided the egg-laying females into the water and prevented them from being devastated outside the river, especially around the street lamps.

## 4. Discussion

Numerous methods have been established to minimize the ecological effects caused by artificial light pollution. For instance, changes in the duration of public lighting can reduce the environmental effects [9,34]. Here we presented a simple method, which provides a useful first step for the effective management of the disruptive influence of artificial light pollution on night-swarming mayflies. Although artificial night-time lighting usually has negative effects on the environment, we managed to use LED lighting beacons for the protection of the nocturnal mayfly, *E. virgo*. Our results imply that any kind of positively phototactic, nocturnal, river-dwelling mayfly species may be saved with our method. Moreover, other dusk- or night-active primary aquatic insects (the larvae and adults of which live in water; e.g. water beetles and bugs) and secondary aquatic insects (the larvae of which develop in water, but the imagoes are terrestrial; e.g. caddisflies (Trichoptera) and non-biting midges (Chironomidae)) also possess positive phototaxis [36]. Thus, our method may also be used to protect these insects during their own swarming period. However, it is a complex question because a strong light source at night for the purpose of saving a small group of insects might have several side effects on other species including fishes, birds and bats. Artificially aggregating mayflies with lights may alter the feeding habits of insectivorous animals, but this effect could be minimized by restricting the beacon activity to mayfly swarming periods.

Previous studies have shown that like the diurnally swarming *Palingenia longicauda* (Olivier, 1791) mayfly [37], the nocturnal *E. virgo* is also positively polarotactic, that is they are attracted to horizontally polarized light [22]. At night, the primary illumination allowing the formation of reflection–polarization patterns of the river surface originates from the direct and sky-scattered moonlight. During their compensatory flight, the females use their polarization sensation to follow the horizontally polarizing track of the river. However, when they encounter a bridge or riverside, the horizontally polarized signal of the river is interrupted by the vertically polarized mirror image of the bridge or riparian vegetation [22,25,38]. Hence, the bridge as an optical barrier is able to interrupt the compensatory flight of both diurnal and nocturnal mayflies. This obstacle leads to decreases in the numbers of individuals and shifting of the sex ratio in *P. longicauda* mayfly populations under and over the bridge [38,39]. But the situation is fatal in the case of the night-swarming *E. virgo*, since the females accumulated in front of the bridge are attracted to the urban lights if present, because their light intensity is much higher than that of the original moonlit environment [22]. Hence, the intensity of the beacon lights should be significantly higher than the average intensity of the surrounding urban lighting, irrespective of the moonlight.

The examples for huge mayfly swarms mentioned in the Introduction occur year after year with varying intensity in spite of the urban lighting being present for decades. Although the length of compensatory flight for *E. virgo* is unknown, according to former assumptions for other species, it may be as long as 3–4 km [40]. It is clear that there are wide stretches of the rivers without distracting illumination further from the urbanized areas where mayfly populations are not disturbed. Thus, our method is relevant mainly at bridges surrounded with illuminated localities. As a side effect, the beacons may reduce the risk of accidents and annoyance caused by mayfly clouds at the affected bridges and may draw the attention of spectators to the beauty of swarming normally hidden in the darkness.

The operating beacons may prevent the female mayflies from continuing their compensatory flight at the bridge because of the high attractiveness of the LED lights. This is, however, not a serious problem, since bridges act as optical barriers for the females [22,38], and our beacon lights just ensure that all of the arriving egg-laying mayflies end up in the river instead of perishing on the dry land or asphalt road under the street lamps. We emphasize that our beacons do not increase permanently the light pollution, because they operate only temporarily, during the few-hour-long swarming period of the concerned mayflies. The appropriate timing of illumination could be predicted from the swarming activities of previous years at a given site, and the beacons could be programmed to operate during nights of a well-defined time period. Moreover, we observed that only *E. virgo* mayflies were rustling around the beacons during our experiments, thus we assume that this conservation method does not distract other species, although it depends on the local fauna, as well.

*Ephoron virgo* has been absent for decades in most of the rivers in central Europe, like the Rhine and Danube. It returned in the 1990s presumably due to an improvement of water quality. It is important to note, that our beacon method may be useless in the case of polluted waters, since water quality is probably the main determinant of mayfly conservation. Thus, this species could be a good bioindicator of ecological quality of rivers [41], and the tributaries could be the sources of recolonization of *E. virgo*. It is therefore essential to protect the mayfly populations of small rivers and the beacons on the bridges could be a successful protective equipment at mayfly aggregation centres.

We successfully tested a conservation method exploiting the positive phototactic behaviour of night-swarming mayflies but it is worth noting that the emission spectrum of the applied beacon lights should be optimized for the spectral sensitivity and phototaxis of the mayflies to be conserved. This could be achieved by electro-retinogram measurements [42] and behavioural tests, but the extremely short lifetime of the imagoes encumbers this task. For example, Horridge *et al*. [43] found that the dorsal eyes of *Atalophlebia* spp. mayflies are purely UV-sensitive and the lateral eyes are most sensitive near 535 nm (green). Meyer-Rochow [42] reported that the eyes of male *Ephemera* mayflies are also maximally sensitive in the UV. It should be noted when choosing the most suitable emission spectrum for the beacons, any risk factors for public health should also be taken into account [44]. On the other hand, the intensity of the beacons used for guiding the females to the water should exceed a critical value depending on the situation, so that the mayflies do not become attracted to the street lamps any longer.

The most important message of our study is that female *E. virgo* mayflies can be prevented from dying outside the river before oviposition has occurred. During the times our beacons were switched on, the numbers of mayflies exiting the river was practically zero. Our results lead us to suggest that, with regard to bridge construction, downstream-facing beacon lights be fixed with the provision of temporal activation.

## Data accessibility

The video clips supporting this article have been uploaded as part of the electronic supplementary material.
